# Predictors of health-related quality of life in Chinese patients receiving treatment for neovascular age-related macular degeneration: a prospective longitudinal study

**DOI:** 10.1186/s12886-020-01561-3

**Published:** 2020-07-16

**Authors:** Wei Bian, Junli Wan, Mingqiong Tan, Jun Su, Yi Yuan, Zonghua Wang, Shiying Li

**Affiliations:** 1 Southwest Hospital/Southwest Eye Hospital, Third Military Medical University (Amy Medical University), Chongqing, 400038 China; 2 Key Lab of Visual Damage and Regeneration & Restoration of Chongqing, GaotanyanStreet 29, Shapingba District, Chongqing, 400038 China; 3 School of Basic Medicine, Third Military Medical University (Army Medical University), Chongqing, 400038 China; 4 School of Nursing, Third Military Medical University (Army Medical University), Gaotanyan Street 29, Shapingba District, Chongqing, 400038 China

**Keywords:** Age-related macular degeneration, Health-related quality of life, Predictor, Longitudinal study

## Abstract

**Background:**

Age-related macular degeneration (AMD) is currently the leading cause of irreversible visual impairment in developed countries and seriously affects the health-related quality of life (HRQoL) of patients. However, the majority of the research in this area employs cross-sectional design; longitudinal research investigating changes in HRQoL and influencing factors is limited. The aim of this study was to use a longitudinal study design to investigate descriptive trends in HRQoL and their predictive factors in Chinese AMD patients receiving treatment with vascular endothelial growth factor inhibitors (anti-VEGF) at baseline and follow-ups.

**Methods:**

In a sample of 142 AMD patients from the outpatient clinic of the Southwest Eye Hospital, a tertiary major hospital in the southwest of China, each patient completed a self-administered questionnaire assessing demographics, clinical features, HRQoL, depression, anxiety, coping style, social support, and self-efficacy at baseline and at 1-, 3-, 6-, and 12-month follow-up appointments.

**Results:**

The total score of HRQoL fluctuated, with the highest score at the 6-month follow-up and the lowest score at baseline. Multivariable linear regression showed the predictors of HRQoL are best-corrected visual acuity (BCVA), income level, depression, and visual acuity (VA) of the treated eye at baseline; BCVA, income, and depression at the 1-month follow-up; duration, area of residence, gender, VA of the treated eye, BCVA, income, anxiety, social support, self-efficacy, and depression at the 3-month follow-up; gender, BCVA, income, anxiety, social support, self-efficacy, depression, negative coping, and positive coping at the 6-month follow-up; and BCVA, social support, self-efficacy, and depression at the 12-month follow-up.

**Conclusions:**

The HRQoL and its predictive factors in Chinese AMD patients receiving anti-VEGF treatment fluctuated over time. It is suggested that medical staff should get more information when planning precise care for improving patients’ HRQoL.

## Background

Age-related macular degeneration (AMD) has become the leading cause of irreversible vision loss and blindness among people over age 50, especially in developed countries [1]. Patients with visual impairment caused by AMD often experience difficulties with daily life, cognitive dysfunction, social isolation, and psychological and emotional disorders, which seriously affects their quality of life [2–4]. Currently, wet age-related macular degeneration (wAMD) is the only form of AMD that can be treated with vascular endothelial growth factor inhibitors (anti-VEGF), which has been shown to be effective in maintaining vision in more than 90% of patients and improving it in 25 ~ 40% of patients [5]. However, 65% of patients were found to have no significant improvement in vision, and 50% were found to have various levels of mental and social disorders [6]. Furthermore, countless invasive intravitreal injections and frequent visits to the eye clinic caused patients to experience pain and stress, even leading to their withdrawal from treatment [7]. A controversy arose as to whether the treatment’s benefit to vision could also improve health-related quality of life (HRQoL). Thus, the HRQoL and influence factors of wAMD patients are becoming a major concern in trying to understand the patients’ real feelings and provide targeted treatment and service.

Recently, an increasing number of studies have been carried out to assess the quality of life of patients with AMD, and significant improvements in HRQoL have been achieved after receiving in travitreal treatment [8–11]. Factors identified as being commonly associated with HRQoL of AMD include female gender [12], best-corrected visual acuity (BCVA), contrast sensitivity, restricted activity days [13], visual acuity, stage of the disease [14], and the number of affected eyes [15]. Since HRQoL changes over time depending on the trajectory and treatment of the disease, few researchers have paid attention to dynamic changes in postoperative HRQoL. In this regard, Finger and Inoue demonstrated that BCVA and HRQoL were significantly improved at the 6-month follow-up and had remained stable at the 12-month follow-up; a change in visual acuity of the treated eye directly influenced the patients’ HRQoL, irrespective of whether the better or the worse eye was treated [9, 16]. In contrast, Wang found better visual function scores were associated with higher overall scores on the National Eye Institute’s Visual Function Questionnaire-25 (NEI VFQ-25) at the3-month follow-up [11]. This change in HRQoL over time was supported by a qualitative study that identified four major themes in living with the disease: cautious optimism, endurance, adaptation, and profound sense of loss [7].

All the literature mentioned above shows that physical, mental, and social functions of AMD patients vary with time after receiving anti-VEGF treatment and that their care needs are constantly changing. However, the vast majority of research in this area employed a cross-sectional design; longitudinal research investigating changes in HRQoL and influence factors is limited. Although a few studies explored the longitudinal changes in the quality of life, their analysis of the influencing factors was based only on demographic data and clinical variables of the patients; little attention was focused on the patients’ psychosocial indicators, such as depression, social support, self-efficacy, which were found to have great impact on HRQoL in our previous studies [17, 18]. Furthermore, there are no longitudinal studies on the predictors of HRQoL in AMD patients receiving anti-VEGF treatment at different time points over a 12-month period. However, in the era of precision medicine, medical staff are required to accurately seek differences among patients, predict their needs at various stages, and provide personalized precise care based on the different stages of the prediction model [19–21]. Fully understanding the positive and negative impact of treatment and AMD patients’ experiences and patterns of adjustment over time when receiving anti-VEGF treatment is needed to establish targeted service delivery and effective interventions to improve the HRQoL.

The aim of the study was to apply a longitudinal study design to investigate descriptive trends in HRQoL and predict factors, from the point of AMD diagnosis to 1 year after receiving anti-VEGF treatment. We will answer the following questions: Are there fluctuations in HRQoL over time after receiving the treatment? What variables predict HRQoL at baseline and at follow-ups?

## Methods

### Participants

Participants were recruited from the outpatient clinic of the Southwest Eye Hospital, a tertiary hospital in southwest China. The inclusion criteria for wAMD patients were: (1) age ≥ 18 years; (2) diagnosis of active exudative AMD with recommended therapy of0.5 mg ranibizumab by the retina specialist;(3) not taking anti-anxiety or anti-depressant medication; and (4) no cognitive impairment.

Participants’ personal and demographic data were collected (name, sex, age, date of birth, marital status, residential area, education, monthly income, and general health status). Information about eye conditions was taken from ophthalmic records at the beginning of the research, including visual acuity, eye affected by AMD, BCVA, visual acuity, and the numbers of intravitreal injections. Visual acuity was tested with Snellen number charts and transformed into LogMAR visual acuity for the analysis.

### Instruments

#### National eye Institute’s visual function questionnaire (NEI VFQ-25)

The NEI VFQ-25 [22] questionnaire was the product of an item-reduction analysis of the 51-item NEI VFQ developed to measure the dimensions of self-reporting vision-related health problems for persons with eye diseases. It contains 25 items and generates 9 subscales: global vision rating, difficulty with near-vision activities, difficulty with distance-vision activities, limitations in social function, vision-specific role difficulties, dependency on others, driving problems, limitations with colour vision, and limited peripheral vision. The options for each item are scored with 0, 25, 50, 75, and 100 points ranging from worst to best. The overall composite score for the NEI VFQ-25 is an average of all items.

#### Centre for Epidemiologic Studies’ depression scale (CES-D)

The CES-D [23] is a 20-item self-rating measurement that includes four subscales: depressed affect, positive affect, somatic symptoms and retarded activity, and interpersonal difficulties. Higher scores indicate a higher degree of depression, scores below 15 points are normal, a score of 16 ~ 19 indicates possible depressive symptoms, and scores above 20 points indicate the presence of depressive symptoms.

#### Hospital anxiety and depression scale (HAD)

The HAD [24] was developed to identify cases (possible and probable) of anxiety disorders and depression among patients in non-psychiatric hospital clinics. It contains an anxiety subscale (HADS-A) and a depression subscale (HADS-D). The scores for each item are measured on a Likert-type scale, and the score for each subscale is the sum of all questions answered. The higher the indicated score is, the higher the level of anxiety or depression.

#### Simplified coping style questionnaire (SCSQ)

The SCSQ [25] is a 20-item questionnaire to assess the coping style of the respondents. Two dimensions of positive and negative coping strategies are addressed in the questionnaire, and each subscale consists of10 items. Each item is rated on a 4-point scale ranging from 0 (*rarely*) to 3 (*always*). Higher scores on the active coping strategies subscale reflect better coping ability, whereas a higher score on the negative coping strategies subscale indicates poorer coping ability.

#### Perceived social support scale (PSSS)

The PSSS [26] consists of 12 items to evaluate a patient’s perception of the social support they receive from family, friends, and significant others. Each item is scored on a 7-point scale ranging from 1 (*very strongly disagree*) to 7 (*very strongly agree*). The overall score is the total score of all items.

#### General self-efficacy scale (GSES)

The GSES [27] is used to test the individual’s general perception of their ability to cope with difficult situations. Ten items are included in the GSES with a 4-point Likert scale ranging from 1 (*not at all true*) to 4 (*exactly true*). The higher the score is, the higher the level of self-efficacy.

### Data collection

The repeated measures design was used in this longitudinal study. Each participant was asked to complete the questionnaires, including demographic data, clinical features, NEI VFQ-25, CES-D, HAD, SCSQ, PSSS, and GSES, in the waiting room of the eye clinic at baseline and follow-ups between December 2015 and February 2018. Baseline data were collected in the first month of diagnosis and subsequently at 1 month, 3 months, 6 months, and 12 months after treatment. Informed written consent was obtained from each participant before joining the study. All participants were informed of the purpose, content, and relevant precautions of the research before entering the group and were assured that they could withdraw from the investigation at any time. Our studies were conducted in accordance with the Declaration of Helsinki. Ethical approval was granted by the Human Ethics Committee of the First Affiliated Hospital, Third Military Medical University (Ethics Reference 2,016,071).

### Statistical analysis

Data analysis was performed with the Statistical Package for the Social Sciences (SPSS, version 20.0). The normal distribution of the data was examined with the Kolmogorov–Smirnov test. Patient characteristics were generalized by mean and standard deviation for normally distributed continuous data, or by median and Inter-Quartile Range for data with skewed distribution; frequencies and percentages were used for categorical data. The possible factors associated with the HRQoL questionnaire were evaluated by univariate analysis. For the normally distributed subscales, the t-test or Pearson’s correlation coefficient was appropriately conducted. For non-normally distributed subscales, the Wilcoxon signed-rank test or Spearman’s rank correlation coefficient was appropriately applied. The relationship between the variables and HRQoL was examined using Spearman’s rank correlation coefficient. Multivariable linear regression analyses were conducted to control for significant impact factors distinguished by the univariate analysis. The two-tailed *P* value < 0.05 was considered statistically significant.

## Results

### Demographics and clinical characteristics

As Table 1 shows, 142 patients with a mean age of 63.49 (SD = 11.07) years were recruited at baseline before their treatment; nearly half (48.59%) were male; the majority (80.28%) were from urban areas compared with 19.72% from rural areas; more than half (56.34%) had a monthly income less than 10,000 RMB; 69.01%were married; 40.85% had tertiary or higher education, and 23.24% had primary or lower education.

**Table 1:** Demographics and clinical characteristics of the patients at baseline and follow-ups

Demographic and clinical features	Number (percentage) Or Mean ± SD				
	Baseline (*N* = 142)	1 Month (*N* = 137)	3 Months (*N* = 131)	6 Months (*N* = 128)	12 Months (*N* = 115)
Age, years (Mean ± SD)	63.49 ± 11.07	63.47 ± 12.11	64.09 ± 18.43	62.89 ± 7.31	63.66 ± 10.03
Gender					
Male	69 (48.59%)	67 (48.91%)	63 (48.09%)	62 (48.44%)	55 (48.59%)
Female	73 (51.41%)	70 (51.09%)	68 (51.91%)	66 (51.56%)	60 (51.41%)
Education					
Primary or lower	33 (23.24%)	32 (23.36%)	30 (22.90%)	29 (22.66%)	24 (20.87%)
Secondary	51 (35.92%)	47 (34.31%)	45 (34.35%)	45 (35.16%)	39 (33.91%)
Tertiary or higher	58 (40.85%)	58 (42.34%)	56 (42.75%)	54 (42.19%)	52 (45.22%)
Marital status					
Married	98 (69.01%)	96 (70.07%)	97 (74.05%)	95 (74.22%)	93 (80.87%)
Not currently married	44 (30.99%)	41 (29.93%)	34 (25.95%)	33 (25.78%)	22 (19.13%)
Area of residence					
Urban	114 (80.28%)	114 (83.21%)	109 (83.21%)	106 (82.81%)	94 (81.74%)
Rural	28 (19.72%)	23 (16.79%)	22 (16.79%)	22 (17.19%)	21 (18.26%)
Income					
≥ 10,000RMB/month	62 (43.66%)	57 (41.61%)	56 (42.75%)	55 (42.97%)	45 (39.13%)
<10,000RMB/month	80 (56.34%)	80 (58.39%)	75 (57.25%)	73 (57.03%)	70 (60.87%)
Eye affected by AMD					
Unilateral cases	112 (68.31%)	109 (79.56%)	107 (81.68%)	106 (82.81%)	93 (80.87%)
Bilateral cases	30 (31.69%)	28 (20.44%)	24 (18.32%)	22 (17.19%)	22 (19.13%)
VA treated eye	0.29 ± 0.03	0.32 ± 0.02	0.38 ± 0.11	0.41 ± 0.18	0.40 ± 0.19
BCVA	0.51 ± 0.12	0.48 ± 0.03	0.58 ± 0.07	0.62 ± 0.21	0.58 ± 0.16
No. of intravitrealinjections		1.02 ± 0.15	3.07 ± 0.25	3.80 ± 0.76	4.70 ± 0.85

In clinical characteristics, 68.31% had only one eye affected by exudative AMD. The mean visual acuity in the treated eye was 0.29 (SD = 0.03), while the mean of best-corrected visual acuity was 0.51 (SD = 0.12). A total of 137 patients were available for follow-up at 1 month, 131 patients were available for 3-month follow-up, 128 for 6-month follow-up, and 115 patients were available for follow-up at 12-month. Sixteen patients dropped out during follow-up examinations because they elected to continue treatment elsewhere. Their absence created no differences in age, sex, education, marital status, area of residence, income, or visual acuity at baseline from those who participated in follow-ups.

### Changes in quality of life over time

**Fig. 1:**
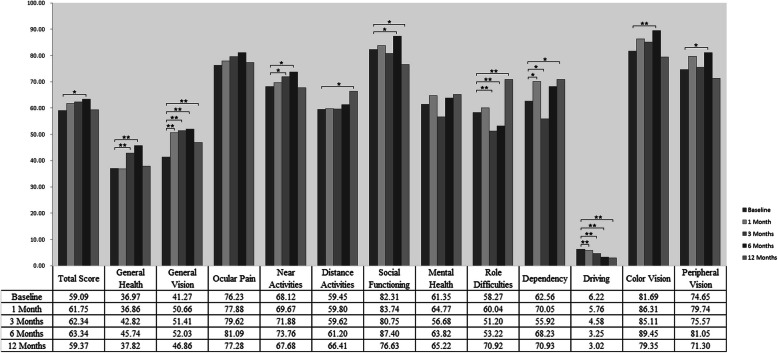
Comparisons of the scores of overall scale and subscales in NEI VFQ-25at baseline and follow-ups

The mean scores of overall scales and subscales at baseline and follow-up are presented in Fig. 1. Comparing the scores at baseline, statistically significant differences were found in three NEI VFQ-25 subscales—general vision, dependency, and driving—at 1-month follow-up after anti-VEGF treatment for AMD; in the scores for six NEI VFQ-25 subscales—general health, general vision, near activities, role difficulties, dependency, and driving—at3-month follow-up; in scores for nine NEI VFQ-25 subscales—total score, general health, general vision, near activities, social functioning, role difficulties, driving, colour vision, and peripheral vision—at 6- month follow-up; and in the scores for six NEI VFQ-25 subscales—general vision, distance activities, social functioning, role difficulties, dependency, and driving—at the 12-month follow-up.

### Predictors of quality of life at follow-ups

All the data were normally distributed; therefore, univariate analyses with t-test or Pearson’s correlation coefficient were performed to find factors that could predict HRQoL (Tables 2 and 3 and Supplementary Table 1). At baseline, the categorical variables marital status (*t* = 5.778, *P <* 0.001), area of residence (*t* = 2.90, *P* = 0.004), income (*t* = 8.06, *P* < 0.001),and eye affected by AMD (*t* = 3.095,*P* = 0.002) were found statistically significant between the t-tested groups. The continuous variables negative coping (*r* = − 0.259, *P <* 0.01), depression (*r* = − 0.270,*P* < 0.01), VA treated eye (*r* = 0.409,*P* < 0.01), and BCVA (*r* = 0.680,*P* < 0.01) correlated significantly with the total score of NEI VFQ-25.

**Table 2:** Comparison of mean NEI VFQ-25 according to categorical variables at baseline and follow-ups

Demographic and clinical features	VFQ-25 score				
	Baseline (*N* = 142)	1 month follow-up (*N* = 137)	3 months follow-up (*N* = 131)	6 months follow-up (*N* = 128)	12 months follow-up (*N* = 115)
Gender					
Male	60.00 ± 15.09 (*n* = 69)	64.68 ± 16.27^*^ (*n* = 67)	57.83 ± 12.03^**^ (*n* = 63)	59.35 ± 13.47^**^ (*n* = 62)	62.26 ± 16.97 (*n* = 55)
Female	58.23 ± 17.54 (*n* = 73)	58.93 ± 15.89 (*n* = 70)	51.12 ± 14.38 (*n* = 68)	53.51 ± 11.16 (*n* = 66)	56.71 ± 19.25 (*n* = 60)
Education					
Primary or lower	55.41 ± 18.99 (*n* = 33)	58.18 ± 19.51 (*n* = 32)	42.83 ± 9.01^**^ (*n* = 30)	54.23 ± 13.46 (*n* = 29)	58.54 ± 19.22 (*n* = 24)
Secondary	57.46 ± 17.51 (*n* = 51)	65.35 ± 15.07 (*n* = 47)	57.26 ± 10.74 (*n* = 45)	57.21 ± 13.20 (*n* = 45)	60.27 ± 15.54 (*n* = 39)
Tertiary or higher	62.62 ± 12.97 (*n* = 58)	60.79 ± 14.93 (*n* = 58)	58.17 ± 13.67 (*n* = 56)	56.74 ± 11.76 (*n* = 54)	59.37 ± 18.33 (*n* = 52)
Marital status					
Married	63.12 ± 17.23^**^ (*n* = 98)	63.37 ± 16.44 (*n* = 96)	54.47 ± 13.67 (*n* = 97)	57.19 ± 12.90 (*n* = 95)	58.34 ± 18.29 (*n* = 93)
Not currently married	50.11 ± 9.4 (*n* = 44)	57.93 ± 15.41 (*n* = 41)	53.98 ± 13.88 (*n* = 34)	53.90 ± 11.64 (*n* = 33)	63.71 ± 18.28 (*n* = 22)
Area of residence					
Urban	61.02 ± 16.64^**^ (*n* = 114)	61.80 ± 14.08 (*n* = 114)	55.15 ± 14.58^*^ (*n* = 109)	57.21 ± 12.90 (*n* = 106)	60.04 ± 18.37 (*n* = 94)
Rural	51.26 ± 12.69 (*n* = 28)	61.47 ± 24.87 (*n* = 23)	50.36 ± 6.67 (*n* = 22)	52.16 ± 10.51 (*n* = 22)	56.33 ± 18.25 (*n* = 21)
Income					
≥ 10,000RMB	69.52 ± 13.64^**^ (*n* = 62)	71.46 ± 13.09^**^ (*n* = 57)	65.02 ± 11.35^**^ (*n* = 56)	60.98 ± 13.69^**^ (*n* = 55)	65.29 ± 16.32^**^ (*n* = 45)
< 10,000RMB	51.01 ± 13.53 (*n* = 80)	54.82 ± 14.76 (*n* = 80)	46.37 ± 9.05 (*n* = 75)	52.84 ± 10.57 (*n* = 73)	55.56 ± 18.64 (*n* = 70)
Eye affected by AMD					
Unilateral cases	61.23 ± 16.05^**^ (*n* = 112)	63.97 ± 15.52^**^ (*n* = 109)	55.63 ± 14.30^**^ (*n* = 107)	56.83 ± 13.14 (*n* = 106)	61.16 ± 17.46^*^ (*n* = 93)
Bilateral cases	51.12 ± 15.26 (*n* = 30)	53.10 ± 16.55 (*n* = 28)	48.62 ± 8.54 (*n* = 24)	53.97 ± 9.71 (*n* = 22)	51.78 ± 20.33 (*n* = 22)

^*^P<0.05,^**^P<0.01

**Table 3:** Correlations between total score of the NEI VFQ-25 and continuous variables at baseline and follow-ups

Variables	NEI VFQ-25 score				
	Baseline	1 month follow-up	3 months follow-up	6 months follow-up	12 months follow-up
Age	0.151	0.147	−0.050	−0.070	0.062
VA treated eye	0.409^**^	0.231^**^	0.586^**^	0.063	0.193^*^
BCVA	0.608^**^	0.419^**^	0.550^**^	0.412^**^	0.443^**^
No. of intravitreal injections		−0.045	− 0.125	0.115	0.136
Anxiety	−0.130	- 0.537^**^	- 0.201*	- 0.363^**^	- 0.341^**^
Depression	−0.270^**^	- 0.533^**^	- 0.237^**^	- 0.798^**^	- 0.656^**^
Positive Coping	0.053	0.068	0.140	0.363^**^	0.048
Negative Coping	−0.259^**^	- 0.075	- 0.001	- 0.328^**^	−0.081
Social Support	0.007	0.165	0.285^**^	0.565^**^	0.427^**^
Self-efficacy	0.051	0.272^**^	0.300^**^	0.336^**^	0.616^**^

^*^P<0.05,^**^P<0.01

At the 1-month follow-up, the categorical variables gender (*t* = 2.092,*P* = 0.038), income (*t* = 6.811,*P* < 0.001), and eye affected by AMD (*t* = 3.260,*P* = 0.001) were found statistically significant between the t-tested groups. The continuous variables anxiety (*r* = − 0.537,*P* < 0.01), depression(*r* = − 0.533, *P* < 0.01), self-efficacy(*r* = 0.272,*P* < 0.01), VA treated eye (*r* = 0.231,*P* < 0.01), and BCVA (*r* = 0.419,*P* < 0.01) correlated significantly with the total score of NEI VFQ-25.

At the 3-month follow-up, the categorical variables gender (*t* = 2.884,*P* = 0.005), education(*t* = 7.337,*P* < 0.001), area of residence(*t* = 2.401,*P* = 0.019), income(*t* = 10.462,*P* < 0.001), and eye affected by AMD (*t* = 3.148,*P* = 0.003) were found statistically significant between the t-tested groups. The continuous variables anxiety (*r* = − 0.201,*P* < 0.05), depression(*r* = − 0.237,*P* < 0.01), social support (*r* = 0.285,*P* < 0.01), self-efficacy(*r* = 0.300,*P* < 0.01), VA treated eye (*r* = 0.586,*P* < 0.01), and BCVA (*r* = 0.550,*P* < 0.01) correlated significantly with the total score of NEI VFQ-25.

At the 6-month follow-up, the categorical variables gender (*t* = 2.679,*P* = 0.008) and income(*t* = 3.796,*P* < 0.001) were found statistically significant between the t-tested groups. The continuous variables anxiety (*r* = − 0.363, *P* < 0.01), depression(*r* = − 0.798,*P* < 0.01), positive coping(*r* = 0.363, *P* < 0.01), negative coping(*r* = 0.328, *P* < 0.01), social support (*r* = 0.565, *P* < 0.01), self-efficacy(*r* = 0.336,*P* < 0.01), and BCVA (*r* = 0.412, *P* < 0.01) correlated significantly with the total score of NEI VFQ-25.

At the 12-month follow-up, the categorical variables income (*t* = 2.866,*P* = 0.005) and eye affected by AMD (*t* = 2.195,*P* = 0.03) were found statistically significant between the t-tested groups. The continuous variables anxiety (*r* = − 0.341,*P* < 0.01), depression(*r* = − 0.656,*P* < 0.01), social support (*r* = 0.427,*P* < 0.01), self-efficacy (*r* = 0.616,*P* < 0.01), VA treated eye (*r* = 0.193,*P* < 0.05),and BCVA (*r* = 0.443,*P* < 0.01) correlated significantly with the total score of NEI VFQ-25.

The total scores of NEI VFQ-25 as the dependent variable along with statistically significant psychosocial variables and covariates were entered into the linear regression analysis (Table 4). At baseline, four covariates (BCVA, income, depression, and VA treated eye) remained significant predictors of NEI VFQ-25 total scores, and the coefficient of determination R^2^ was 0.559. At the 1-month follow-up, two covariates (BCVA and income) and one psychosocial variable (depression) remained significant predictors of NEI VFQ-25 total scores, and the coefficient of determination R^2^ was 0.403. At the 3-month follow-up, six covariates (education, area of residence, gender, VA treated eye, BCVA, and income) and four psychosocial variables (anxiety, social support, self-efficacy, and depression) remained significant predictors of NEI VFQ-25 total scores, and the coefficient of determination R^2^ was 0.833. At the 6-month follow-up, three covariates (gender, BCVA, and income) and six psychosocial variables (anxiety, social support, self-efficacy, depression, negative coping, and positive coping) remained significant predictors of NEI VFQ-25 total scores, and the coefficient of determination R^2^ was 0.748. At the 12-month follow-up, two covariates (BCVA and eye affected by AMD) and three psychosocial variables (social support, self-efficacy, and depression) remained significant predictors of NEI VFQ-25 total scores, and the coefficient of determination R^2^ was 0.509.

**Table 4:** Linear regression analysis of predictors of quality of life at baseline and follow-ups

Variables	UnstandardizedCoefficientsBeta	Std.Error	Standardized CoefficientsBeta	t	***P*** value	95% Confidence interval for B	
						Lower Bound	Upper Bound
**Baseline**							
(Constant)	79.843	6.135		13.015	.000	67.712	91.975
BCVA	24.499	3.494	.458	7.012	.000	17.589	31.408
Income	−8.652	2.204	−.263	−3.926	.000	−13.010	−4.294
Depression	−7.454	2.304	−.182	−3.235	.002	−12.010	−2.898
VA treated eye	4.290	2.119	.122	2.025	.045	8.480	101.000
**1 Month follow-up**							
(Constant)	84.703	6.122		13.836	.000	72.590	96.815
BCVA	11.851	3.651	.237	3.246	.001	4.627	19.075
Income	−7.287	2.387	−.228	−3.052	.003	−12.011	−2.563
Depression	−.529	.235	−.231	−2.247	.026	−.994	−.063
**3 Months follow-up**							
(Constant)	60.453	6.748		8.958	.000	47.092	73.815
Anxiety	−.732	.100	−.495	−7.288	.000	−.931	−.533
VA treated eye	34.764	5.160	.551	6.738	.000	24.549	44.980
Social Support	.278	.053	.272	5.216	.000	.384	.173
Self-Efficacy	.684	.136	.316	5.034	.000	.415	.953
Income	−6.337	1.399	−.230	−4.530	.000	−9.106	−3.567
Education	3.158	.702	.182	4.497	.000	1.767	4.548
Depression	−.637	.188	−.279	−3.383	.001	−.264	−1.010
BCVA	11.508	3.811	.231	3.020	.003	19.053	3.963
Area of residence	−4.835	1.906	−.133	−2.537	.012	−1.061	−8.609
Gender	−2.786	1.151	−.102	−2.422	.017	−5.064	−.508
**6 Months follow-up**							
(Constant)	105.511	11.282		9.352	.000	83.169	127.854
Depression	−1.974	.179	−1.690	−11.025	.000	−2.329	−1.620
Anxiety	1.902	.275	.945	6.910	.000	1.357	2.447
Negative coping	−1.397	.270	−.325	−5.175	.000	−1.931	−.862
Social Support	.430	.096	.275	4.467	.000	.240	.621
Positive coping	.823	.189	.243	4.350	.000	.448	1.198
Gender	−6.563	1.553	−.261	−4.227	.000	−9.638	−3.488
Self-Efficacy	−.606	.141	−.264	4.307	.000	−.885	−.328
BCVA	14.622	3.728	.247	3.922	.000	22.004	7.240
Income	−3.466	1.439	−.136	−2.408	.018	−6.316	−.615
**12 Months follow-up**							
(Constant)	26.270	10.321		2.545	.012	5.814	46.726
Self–Efficacy	1.082	.187	.424	5.785	.000	.711	1.452
BCVA	19.478	4.658	.285	4.181	.000	10.245	28.710
Depression	−.316	.114	−.196	−2.759	.007	−.542	−.089
Eye affected by AMD	−8.737	3.216	−.188	−2.717	.008	−15.112	−2.363
Social Support	.215	.093	.173	2.302	.023	.030	.400

## Discussion

By monitoring the HRQoL in patients with wAMD receiving anti-VEGF treatment from baseline through their periodic follow-ups for 1 year, two significant findings were identified. First, the scores of the NEI VFQ-25 in overall score and subscales fluctuated before and after wAMD patients received treatment, with the highest scores at the 6-month follow-up and lowest at the baseline. Second, the variables that predict HRQoL varied across time, with only BCVA and depression remaining as predictors across all five time points.

In our study, total score and the subscales related to vision (i.e., general vision, general health, near activities, distance activities, colour vision, and peripheral vision) on the NEI VFQ-25 had improved over the course of treatment and reached their peaks at the 6-month follow-up. This may imply that the continuous improvement of vision and wAMD patients’ daily living abilities in the first 6 months improved their HRQoL with time. However, in the present study, the total HRQoL score at the 12-month follow-up had dropped to the same position as the baseline, which is in line with Finger’s findings [16]. A low number of injections and irregular follow-ups caused unstable improvement of vision; high expectations and disappointment with treatment outcomes may lead to HRQoL’s improvement not being maintained for12 months. Furthermore, due to fewer professional vision rehabilitation institutions and an incomplete service network, the need for visual rehabilitation of the patients could not be met in China at present. Compared to a developed country, it is impossible to provide more comprehensive information support, supervision of regular follow-ups, and professional visual function training for discharged patients as in a developed country [28]. In the meanwhile, the social functioning subscale followed the variation trends of the subscales related to vision. The patients were more willing and open to communicating with others and participating in social activities after their vision had improved. Otherwise, they preferred to stay at home and isolate themselves from the outside world. We also found that the scores on the driving subscale constantly declined from the baseline through the follow-ups. This result may be explained by the fact that driving requires a high level of vision, so most wAMD patients had given up driving, regardless of whether their vision improved after treatment. One interesting finding is that the scores of the subscales of role difficulties and dependency were lowest at the 3- and 6-month follow-ups, although their vision had significantly improved. A possible explanation for this may be that most wAMD participants were elderly people, and by Chinese tradition, they are taken care of by their spouses or children rather than in professional institutions. Relatives played an important role in their vision rehabilitation. They typically help patients with daily activities, give comfort, and take them to return visits, which were most frequent in the first 6 months, so the patients perceived more dependency on their families [29]. On the other hand, increased dependence would diminish the initiative and authority of the elderly at home, causing a strong sense of loss, which could lead to their difficulty with role adjustment.

The subsequent step aimed to ascertain which variables predict HRQoL at different time points. With the total score of NEI VFQ-25 as the dependent variable, the regression analysis was carried out with 17 variables in the demographic data, medical condition, and social psychological index as the independent variables. The results showed that the HRQoL in wAMD patients was influenced by many factors that changed along the course of treatment.

Demographics like gender, area of residence, education, and income were identified as significantly influencing HRQoL. Male patients with high income and education levels who live in urban areas showed better HRQoL. At the early and middle stages of treatment, the economic burden was an important factor affecting quality of life. Due to the Chinese health care system, wAMD patients have to cover expensive anti-VEGF drugs at their own expense, which becomes a burden to the family. Therefore, lower income means a heavier economic burden and thus, poorer quality of life. In the latter stages of treatment, with the decrease in the frequency of treatment, the patient’s financial burden is reduced, and the economic burden ceases to be a main factor affecting the quality of life of patients at the 12-month follow-up.

The area of residence and education played an important role in predicting HRQoL at the 3-month follow-up. During the first 3 months of treatment, a lack of knowledge of the disease was an important factor affecting the quality of life. Patients with higher educational levels were more inclined to effectively seek information support [30]. During the course of treatment, patients with lower educational levels began to acquire more information about the disease, and the impact of education on the quality of life of patients declined. On the other hand, gender was found to influence the HRQoL at the 3- and 6-month follow-ups. The total NEI-VFQ-25 score of female patients was lower than that of male patients. The reason for this may be that the personality characteristics of female patients are vulnerable and sensitive, and negative emotions such as nervousness, depression, and inferiority are more likely to occur than in male patients, resulting in poorer quality of life [31].

In addition to demographic variables, we also found that clinical characteristics such as BCVA and VA treated eye were significant factors predicting HRQoL. The improvement in BCVA was associated with improved HRQoL, which has been proven in many studies [32, 33]. A possible explanation is that patients relied on BCVA for their daily activities through the whole process of treatment, and could get better use of residual vision with the rehabilitation training [34]. Therefore, BCVA became the key impact factor to serve as a predictor across the five time points. On the other hand, patients paid greater attention to the VA treated eye in the early and middle stages of treatment, then adapted to the changes of visual function, and the visual acuity of the treated eye was no longer an important factor of HRQoL at the 12-month follow-up. Also, the impact factor “eye affected by AMD” was found to influence the HRQoL at the 12-month follow-up. NEI VFQ-25 scores of bilateral patients were lower than those of unilateral patients [35]. This may be because patients are mainly concerned with BCVA and VA treated eye in the early stage. When the vision is stable or weakening, unilateral patients can still depend on the visual acuity of the unaffected eye, while bilateral patients are less able to adapt to losing their visual function in the later stage [31].

Consistent with other studies, we found higher depression and anxiety were significantly associated with poorer HRQoL [12, 36], and that depression was found to be a predictor of HRQoL across all time points. The prevalence of depression in AMD has been reported as ranging from 17.9% [37] to 43% [38]. Symptoms of AMD, including difficulty in daily activities, recognising others, and participating with housework, may lead directly to social isolation, dependence on others, and role conflicts which would in turn result in increased depression and anxiety [2]. Also, because of the uncertainty of AMD treatment, they may constantly worry over the whole treatment period about whether the positive effects of treatment can be sustained or the disease will eventually lead inevitably to blindness [7]. Furthermore, the great economic burden would aggravate the symptoms of depression and anxiety, especially in the middle stage of treatment. However, some patients tried to manage daily life difficulties and emotional conflict by developing positive coping strategies and taking a positive, optimistic attitude towards life during the longer duration of AMD. Therefore, the symptoms of anxiety and depression eased in later stages.

Social support was found to be significantly related to HRQoL at 3-, 6-, and12-month follow-ups. Social support was confirmed to help patients promote a good mental state and face diseases caused by a variety of physical functions and psychological and social difficulties, and to improve the patients’ compliance and quality of life [39]. In the Chinese culture, family is the main source of social support during long-term treatment and rehabilitation. They were involved in the vision rehabilitation services, which had a vital impact on the health outcomes of patients. Furthermore, support like information on how to manage the symptoms, improve one’s capacity for psychological adjustment, and a management strategy supplied by the medical staff were an important factor in promoting improvement of survival quality [40].

We assessed the effect of self-efficacy and found it has a positive effect on quality of life at 1-, 3-, and 6-month follow-ups. Self-efficacy refers to the extent to which people believe their actions will lead to a certain outcome, which means the expectation that an individual can successfully perform a certain behaviour [41]. With the treatment’s progression, patients with a high level of self-efficacy regarded conflicts and difficulties as a chance to improve their abilities and had greater confidence in the success of the treatment. They imagined the success scenario and adopted positive health behaviours to promote the effective cognitive reconstruction process. Similarly, a healthy and effective cognitive and behavioural experience can strengthen self-efficacy beliefs, so these patients would show more active adaptability and strive to overcome the various symptoms caused by daily life and social and psychological barriers throughout the treatment; the nursing process could improve the HRQoL of the AMD patient [42].

Other psychosocial variables, such as negative and positive coping, were demonstrated to be significant determinants of HRQoL at the 6-month follow-up, which was partly consistent with Sturrock’s findings [43]. In their study, only avoidance coping, not acceptance coping, was observed to significantly determine a decline in vision-related functioning. This may be due to inadequate organization of professional vision rehabilitation to help patients develop positive coping ability in China. Patients had to formulate special coping strategies gradually when dealing with the disease. Therefore, positive or negative coping style will affect patients’ subjective understanding, problem-solving ability, and mental health. Also, different scales used to test coping ability may have led to different outcomes.

As with all studies, certain limitations must be noted. First, a relatively small and uniform sample was recruited in our study, so the findings may not be generalized to all AMD patients in China. Second, due to time constraints and patient dropouts, we completed only the 12-month follow-up. An advanced computer-based HRQoL instrument like an eye-term bank [44] should be adopted in future studies to evaluate the predictors of HRQoL changes. Finally, although we tried our best to include all the impact factors that we assumed to be predictors of HRQoL in AMD patients, other potential predictors may have been missed that could be significantly related to HRQoL. Nevertheless, this study’s focus was on HRQoL and predictors of disease trajectory over five time points, which could elucidate its dynamic changes more precisely and provide more accurate information for clinical work. Not only sociodemographic and clinical characteristics but also psychosocial indicators that influence HRQoL were brought into our predicting analysis, which had been neglected or incomplete in other studies. Future studies should compare the differences in the HRQoL scores of patients receiving different protocols and drugs.

## Conclusions

As hypothesized, the scores and predictors of HRQoL of Chinese patients with AMD receiving anti-VEGF treatment fluctuated over time. Therefore, understanding the changing role of the predictors at different stages of treatment assists medical staff by providing more information to guide their planning of targeted intervention and supportive care to improve the HRQoL of AMD patients at different times in their treatment. At the early stage, greater attention should be paid to low-income patients and those with limited visual acuity. As depression has been a predictor at all stages of treatment, psychosocial intervention and sufficient information support should be provided to decrease the incidence of depression [45, 46]. At the middle stage of treatment, special attention should be given to the various discomforts, anxiety, and depression caused by the treatment, especially in female patients from rural areas with lower levels of education and income. Coping strategies and self-management should be taught and mastered to face the difficulties caused by the disease and increase self-efficacy. Family members should be encouraged to participate in presenting treatment procedures and rehabilitation. Finally, visual function training should be strengthened to maximize the use of residual vision if visual function cannot be improved or sustained at the later stages. In this way, patients can get comprehensive, integrated, and precise care at different stages of treatment and improve their HRQoL in long-term outcomes.

## Supplementary information

**Additional file 1: Table S1.** Mean scores of questionnaires of CES-D, HAD, SCSQ, PSSS and GSES at baseline and follow-ups

## Data Availability

The datasets used and/or analysed during the current study are available from the corresponding author on reasonable request.

## Abbreviations

AMD: Age-related macular degeneration
wAMD: wet age-related macular degeneration
HRQoL: Health-related quality of life
anti-VEGF: vascular endothelial growth factor inhibitors
BCVA: Best-corrected visual acuity
NEI-VFQ-25: National Eye Institute Visual Function Questionnaire
CES-D: Center for epidemiologic studies depression scale
HAD: Hospital Anxiety and Depression Scale
SCSQ: Simplified coping style questionnaire
PSSS: Perceived Social Support Scale
GSES: General Self-Efficacy Scale
