# Supplementary material for: Predictors of health-related quality of life in Chinese patients receiving treatment for neovascular age-related macular degeneration: a prospective longitudinal study
Source: BMC Ophthalmol. 2020 Jul 16;20:291. doi: 10.1186/s12886-020-01561-3 (PMC7364534; doi:10.1186/s12886-020-01561-3)
Supplement: Supplementary file 1 — Additional file 1: Table S1. Mean scores of questionnaires of CES-D, HAD, SCSQ, PSSS and GSES at baseline and follow-ups [file 12886_2020_1561_MOESM1_ESM.docx]

**Supplementary Table 1 Mean scores ofquestionnairesof CES-D, HAD, SCSQ, PSSS and GSES at baseline and follow-ups**

| Variables | Baseline | 1 month  follow-up | 3 month  follow-up | 6 month  follow-up | 12 months  follow-up |
| --- | --- | --- | --- | --- | --- |
| Anxiety | 33.65±7.57 | 34.33±9.08 | 34.26±9.07 | 15.64±6.27 | 37.98±11.36 |
| Depression | 14.85±4.57 | 16.65±6.83 | 16.62±6.85 | 33.41±10.81 | 13.70±8.11 |
| Positive Coping | 19.93±5.13 | 22.37±6.97 | 22.29±6.95 | 21.85±3.72 | 22.49±6.43 |
| Negative Coping | 8.44±2.77 | 8.93±3.13 | 8.97±3.11 | 11.01±2.94 | 10.82±4.42 |
| Social Support | 60.50±11.50 | 61.99±11.61 | 61.91±11.62 | 59.20±8.07 | 62.51±14.80 |
| Self-efficacy | 24.63±5.03 | 26.35±6.00 | 26.39±6.00 | 27.21±5.50 | 28.40±7.19 |
